# Overnight variation in tidal expiratory flow limitation in COPD patients and its correction: an observational study

**DOI:** 10.1186/s12931-021-01913-7

**Published:** 2021-12-23

**Authors:** J. McKenzie, P. Nisha, S. Cannon-Bailey, C. Cain, M. Kissel, J. Stachel, C. Proscyk, R. Romano, B. Hardy, P. M. A. Calverley

**Affiliations:** 1 Philips Respironics, Monroeville, PA USA; 2 Institute of Ageing and Chronic Disease, University of Liverpool, Liverpool, UK; 3 University Hospital Aintree, Longmoor Lane, Liverpool, L23 8UE UK

**Keywords:** COPD, Noninvasive ventilation (NIV), Tidal expiratory flow limitation (EFL_T_), Forced oscillation technique, Expiratory positive airway pressure (EPAP)

## Abstract

**Background:**

Tidal expiratory flow limitation (EFL_T_) is common among COPD patients. Whether EFL_T_ changes during sleep and can be abolished during home ventilation is not known.

**Methods:**

COPD patients considered for noninvasive ventilation used a ventilator which measured within-breath reactance change at 5 Hz (∆Xrs) and adjusted EPAP settings to abolish EFL_T_. Participants flow limited (∆Xrs > 2.8) when supine underwent polysomnography (PSG) and were offered home ventilation for 2 weeks. The EPAP pressure that abolished EFL_T_ was measured and compared to that during supine wakefulness. Ventilator adherence and subjective patient perceptions were obtained after home use.

**Results:**

Of 26 patients with supine EFL_T_, 15 completed overnight PSG and 10 the home study. In single night and 2-week home studies, EFL_T_ within and between participants was highly variable. This was unrelated to sleep stage or body position with only 14.6% of sleep time spent within 1 cmH_2_O of the awake screening pressure. Over 2 weeks, mean EPAP was almost half the mean maximum EPAP (11.7 vs 6.4 cmH_2_O respectively). Group mean ∆Xrs was ≤ 2.8 for 77.3% of their home use with a mean time to abolish new EFL_T_ of 5.91 min. Adherence to the ventilator varied between 71 and 100% in prior NIV users and 36–100% for naïve users with most users rating therapy as comfortable.

**Conclusions:**

Tidal expiratory flow limitation varies significant during sleep in COPD patients. This can be controlled by auto-titrating the amount of EPAP delivered. This approach appears to be practical and well tolerated by patients.

*Trial registration*: The trial was retrospectively registered at CT.gov NCT04725500.

**Supplementary Information:**

The online version contains supplementary material available at 10.1186/s12931-021-01913-7.

## Background

Tidal expiratory flow limitation (EFL_T_) occurs during quiet breathing when flow cannot be increased without an increase in end-expiratory lung volume (EELV). As COPD progresses, tidal expiratory flow limitation (EFL_T_) develops and contributes to dynamic hyperinflation and its associated intrinsic positive end-expiratory pressure (PEEPi)) at rest and during exercise [1, 2]. The presence of EFL_T_ measured when seated is associated with more dyspnoea on exertion [3] and a greater chance of exacerbation and hospitalisation in patients with moderate to severe COPD [4]. The likelihood of developing EFL_T_ is markedly increased by postural change when EELV falls [5, 6]. Lung volume falls further in a state-dependent fashion with the onset of sleep [7, 8], but to date, we lack data about how this impacts EFL_T_.

Noninvasive ventilation (NIV) is an effective strategy for the management of chronic hypercapnic COPD [9]. Different ventilatory approaches have been adopted with high-pressure ventilation proving effective in reducing exacerbations in these sick patients [10]. The application of suboptimal or excessive PEEP may negatively impact adherence to NIV and may result in patient non-compliance [11]. Expiratory flow limitation can be a problem for the ventilated COPD patient and contributes to poor patient-ventilator coordination [12]. Although bronchodilators can abolish EFL_T_ in some COPD patients [13], this can be reliably achieved only by the application of sufficient end-expiratory pressure (EPAP) with a resultant decrease in the drive to breathing [14]. Manual pressure titration and clinical judgement usually determine the EPAP which is typically set at a fixed value. However, if EFL_T_ is variable, a fixed EPAP pressure may be inadequate or excessive at any given time reducing the clinical benefit of NIV [15]. EFL_T_ can be accurately identified noninvasively using the method of Dellaca et al. based on the within breath change in respiratory system reactance using the forced oscillation technique (FOT) at 5 Hz (X_5_) [16]. This approach has been adapted to provide continuous monitoring of EFL_T_ during noninvasive ventilation [17]. Recently these investigators have shown that changing EPAP can abolish EFL_T_ monitored on a breath by breath basis, that this system works in different postures and that it can have physiological benefits in COPD patients studied for one night [18]. However, there is no reported experience with longer periods of ventilatory support using this system.

Before determining whether this new approach is more effective in providing long-term ventilatory support in COPD patients, we conducted an observational study in COPD patients with established tidal expiratory flow limitation when supine, first to determine whether variation in EFL_T_ during sleep irrespective of posture and second whether overnight EFL_T_ could be abolished by this novel method of ventilatory support in a clinically acceptable way over an extended period.

## Methods

This was a prospective, observational open-label study. Participants were recruited from a convenience sample of patients with a diagnosis of COPD based on standard criteria [19] from two independent pulmonary practices. Participants gave written, informed consent for the study, which was approved by Allendale Investigational Review Board. The study was retrospectively registered at CT.gov NCT04725500. As these were pilot studies, no formal sample size or power calculations were performed.

### Participant entry criteria and exclusions

Participants between the ages of 40 and 80 with a prior COPD diagnosis either using or being considered for ventilatory support were screened for study inclusion. All participants were clinically stable and able to maintain a SpO2 greater than 88% at rest and during EPAP titration. Patients with OSA/COPD overlap were also considered eligible provided their OSA was controlled by appropriate inspiratory pressure support. A full list of inclusion and exclusion criteria are provided in the Additional file 1.

### Study design

We used the proprietary ExpiraFlow technology delivered by a noninvasive ventilator (BiPAP A40 EFL, Philips/Respironics, Monroeville, PA) first to determine the presence of EFL_T_ awake and then when supine during sleep. In brief, this device uses the forced oscillation methodology to record respiratory system reactance at 5 Hz during tidal breathing on a breath-by-breath basis, partitioned into inspiratory and expiratory phases as previously described [16]. Further information about the modification of this methodology in this ventilator and details of breath selection and rejection for the calculation of the within-breath reactance change are provided in the online data supplement. We chose a threshold of within-breath reactance change (ΔXrs) of more than 2.8 cmH_2_O/L/s averaged over 20 artefact-free breaths to identify the presence of EFL_T_. When used in ventilatory mode, the BiPAP A40 EFL monitors ΔXrs on a breath-by-breath basis. When EFL_T_ occurs the ventilator automatically increases the EPAP level until EFL_T_ is abolished and PEEPi reduced by applying and adjusting pressure dynamically per-flow limited breath [17] (ExpiraFlow™ ventilation technology, Philips Respironics). This approach has been shown to substantially reduce the work of breathing in COPD patients breathing spontaneously and with oral-nasal CPAP [16, 20].

The study had 3 phases. In the first phase, we identified individuals with EFL_T_ when seated and supine using the ExpiraFlow technology with 3 cm H_2_O EPAP delivered with an oro-nasal mask (Comfort Gel Full Face Mask, Philips Respironics). During the 5-min screening, we needed between 5 and 20 technically valid breaths to determine whether flow limitation was present (see Additional file 1 for details of breath selection) If the presence of flow limitation was confirmed, the ExpiraFlow technology automatically adjusted EPAP to abolish EFL_T_ over a 20 min period of supine breathing and that EPAP value was recorded. The mean pressure needed to overcome EFL_T_ in both positions during the titration session was noted as “clinical therapy screening EPAP”.

In the second phase, only participants with EFL_T_ when supine in phase 1 underwent a further 20 min session using the ventilator in S/T mode with EPAP starting at 4 cm H_2_O and pressure support of 6 cm H_2_O together with the FOT. Six cmH_2_O of pressure support was used to ensure that participants had established and EFL_T_ and to allow for potential variability in the severity of EFL_T_ during the night, thereby avoiding a ‘’floor effect when the EPAP could not be reduced further. Again, the ExpiraFlow technology automatically titrated EPAP until EFL was abolished when awake and supine. If the EPAP that abolished EFL_T_ was ≥ 6 cmH_2_O in NIV naïve participants or at least equal to the participant’s prescribed positive airway pressure (determined outside of the study to address any OSA or oxygenation concerns) for experienced NIV patients, the participant underwent overnight polysomnography (PSG) in a sleep laboratory following current AASM recommendations [21] with the ventilator set in the S/T mode as described above. EPAP was automatically adjusted throughout the night to counteract EFL_T_. PSG data were initially scored by an automated scoring system (Somnolyzer Philips Respironics) and were verified and scored manually by an accredited RSGPT.

In the third phase, participants completing the PSG were asked if they were willing to use the ventilator each night for a 2-week in-home study. Therapy data were saved on the ventilator’s internal memory SD card and were analyzed using Philips proprietary software.

### Data analysis and study outcomes

Tidal expiratory flow limitation was considered to be a binary state. i.e. it was present (∆Xrs > 2.8 cmH_2_O/L/s) or absent. EPAP and ∆Xrs values were collected and analyzed. The data we report as EPAP is equivalent to automatically titrated PEEPopt [18] i.e. the end-expiratory pressure required to abolish tidal expiratory flow limitation. The overnight variation in EFL_T_ was identified by the change in auto-titrated EPAP during sleep while using the ventilator. Abolition of EFL_T_ was deemed to be achieved when the average ∆Xrs was < 2.8 cmH_2_O/L/s over a 2 min period.

In phase 2 our primary interest was to determine whether the observed EPAP, our marker of the presence of EFLt, differed during PSG-confirmed sleep from the mean EPAP needed to abolish flow limitation during supine wakefulness. We report data about sleep quality and, where available, the influence of posture and spirometric variables on this outcome. The polysomnographic data were used to determine sleep quality in the single night study and were not related directly to changes in EFL_T_.

In phase 3 we wished to establish the minimum, maximum and mean EPAP values during the 2 week study period together with objective adherence data as the average number of hours of treatment use per day and the patients subjective impressions of treatment.

### Statistical analysis

Data are presented as mean and standard deviation or IQR as appropriate. In the single-night study, the percentage of treatment time in which the EPAP pressure averaged over 2 min was higher, lower or equivalent to the clinical screening EPAP was calculated and presented graphically.

For each participant, the mean values calculated for EPAP are the mean for the days of usage over the two week in-home device use. The mean maximum EPAP value is the mean of the highest EPAP required to abolished EFL on each study night. Additionally, the mean 90% EPAP value is the mean value of EPAP over the 2-week study period at which the participant spent 90% of their time at or below. EPAP and ∆Xrs values were collected and analyzed to investigate trends using a moving average and linear regression techniques. Sleep quality, sleep staging and body position data are presented using descriptive statistics.

## Results

**Fig. 1:**
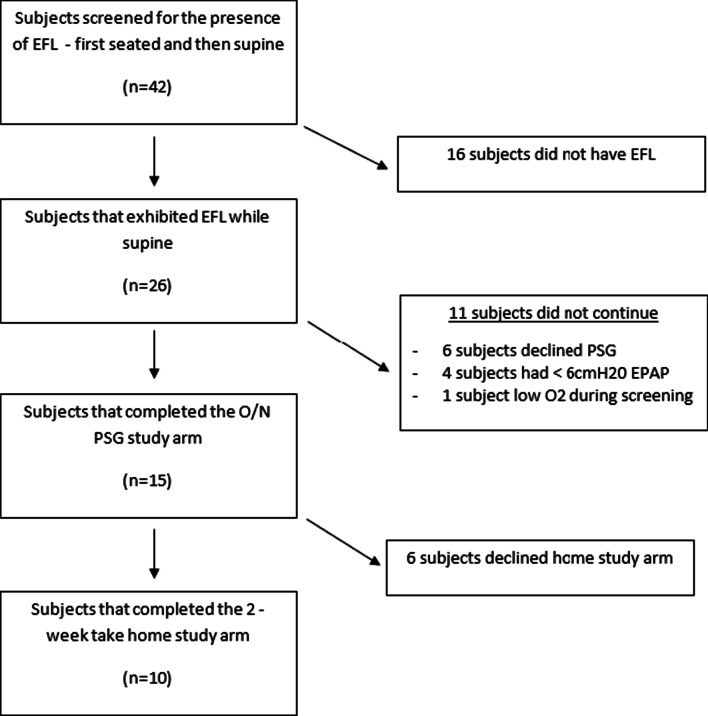
Study participants flow diagram. *Refers to patient 16 who had EFL_T_ when screened but was unable to complete the PSG study night due to acute illness

Participant disposition is summarized in Fig. 1. Forty-two participants previously diagnosed with mild to severe COPD consented to be in the study. Twenty-three of these participants were prior CPAP or bilevel positive airway pressure users. Twenty-six participants exhibited EFL_T_ while supine and four of these participants also exhibited EFL_T_ when seated.

Of the 26 participants with EFL_T_ while supine, 16 were eligible for the overnight PSG and two week in-home study (56% male, mean (SD) age 64.9 (6.2) years, BMI 30.3 (7.01). FEV_1_ (% predicted) 47.2 (10.3) and FEV_1_/FVC (%) 55 (16.9) (Table 1). Fifteen participants agreed to the overnight PSG (Table 1). Ten participants went on to the in-home study. During the in-home study, the two participants with diagnosed obstructive sleep apnoea had their minimum EPAP set to their current CPAP or EPAP settings, if greater than 4 cm H_2_O. The overnight studies did not adjust pressures for any upper airway events.

**Table 1:** Demographic, physiological and observational data for all subjects that completed the overnight PSG study or the 2-week in-home ventilator use study

Participant #	Sex	Age	BMI	FEV1 (% predicted)	FEV1/FVC	Sp02	Previous ventilation user	Overnight PSG	2-Week take home
1	M	70	32.4	39	52	92	No	Yes	Yes
2	F	64	30.2	53	68	95	No	Yes	Yes
3	M	68	30.5	48	49	93	No	Yes	No
4	F	78	24.8	33	33	95	No	Yes	No
5	M	71	38.7	32	32	91	No	Yes	No
6	M	62	39.7	67	86	97	Bilevel	Yes	No
7	M	53	25.9	47	61	94	CPAP	Yes	No
8	F	65	27.1	56	56	94	No	Yes	Yes
9	F	68	35.6	43	38	93	No	Yes	Yes
10	M	69	28.9	54	72	93	Bilevel	Yes	Yes
11	F	63	21.6	35	45	96	Bilevel	Yes	Yes
12	M	56	14.5	48	60	90	No	Yes	Yes
13	M	67	31.5	40	52	95	Bilevel	Yes	Yes
14	M	64	42.3	52	42	92	Bilevel	Yes	Yes
15	F	64	28.6	45	45	97	No	Yes	No
16	F	57	33.56	65	88	97	CPAP	No*	Yes
Average		64.9	30.3	47.2	55	94			
Std. Dev		6.2	7.01	10.3	16.9	2.1			

^*^This patient had to withdraw from the PSG night due to influenza but was keen to complete the study when they recovered from the acute illness

### Single night ventilator study with overnight polysomnography

**Fig. 2:**
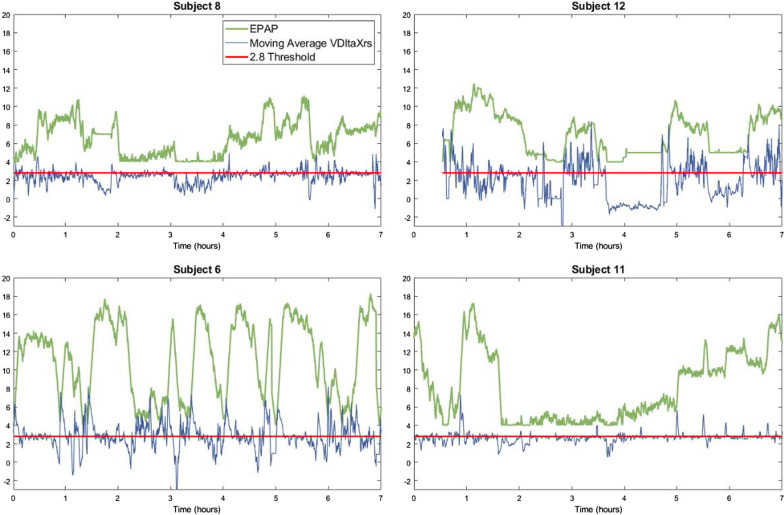
Four representative cases showing overnight EFL_T_ variability and the counteracting EPAP dynamically responding to abolish EFL_T_ and bring ∆Xrs values < 2.8 EPAP adjustments (green lines) during the PSG are plotted against a moving average of ∆Xrs data (blue lines). The threshold ∆Xrs of 2.8 (red line) indicates the presence of EFL_T_ (EFL_T_ = ∆Xrs > 2.8 cmH_2_O/L/s). ∆Xrs values < 2.8 indicates the absence of EFL_T_

Overnight sleep quality varied significantly, as seen in Table 2. One ventilator-naïve participant did not sleep and technical difficulties meant that PSG data from participant 4 were not available. We observed significant between-subject variability in EFL_T_. Figure 2 presents the variability of EPAP and EFL_T_ throughout the night in four representative cases (participants 8 and 12 naïve to NIV, participants 6 and 11 experienced NIV users) Participants 8 and 11 had ∆Xrs values close to the 2.8 cmH_2_O/L/s threshold throughout the night. In contrast, ∆Xrs shows large fluctuations throughout the night in participants 12 and 6 with a similar response by the ExpiraFlow technology to adjust EPAP.

**Table 2:** Individual sleep data obtained during the single night polysomnogram

Participant ID	1	2	3	4	5	6	7	8	9	10	11	12	13	14	15
AHI (avg/h)	26	26	67.5	NA	40.6	5.9	9	3.8	1	5	0	12	0	2	19
Time in wake stage (min)	93	38	394	NA	91	263	210	60	294	325	158	219	228	135	208
Time in N1 stage (min)	22	19	6	NA	30	61	43	43	26	52	14	50	15	7	23
Time in N2 stage (min)	123	232	2	NA	188	124	145	145	62	54	193	172	94	130	113
Time in N3 stage (min)	0	0	0	NA	0	6	32	83	35	0	38	0	42	108	0
Time in REM stage (min)	47	126	0	NA	35	3	37	90	78	47	20	48	36	9	14
Total recording time (min)	285	416	402	NA	345	458	467	421	496	479	423	490	415	391	358
Total sleep time (min)	192	377	8	NA	254	195	258	361	202	154	265	270	187	256	150
Sleep efficiency (%)	67%	91%	2%	NA	74%	42%	55%	86%	41%	32%	63%	55%	45%	65%	42%

Study number represents the same individuals in Table 1. *NA* not available

**Fig. 3:**
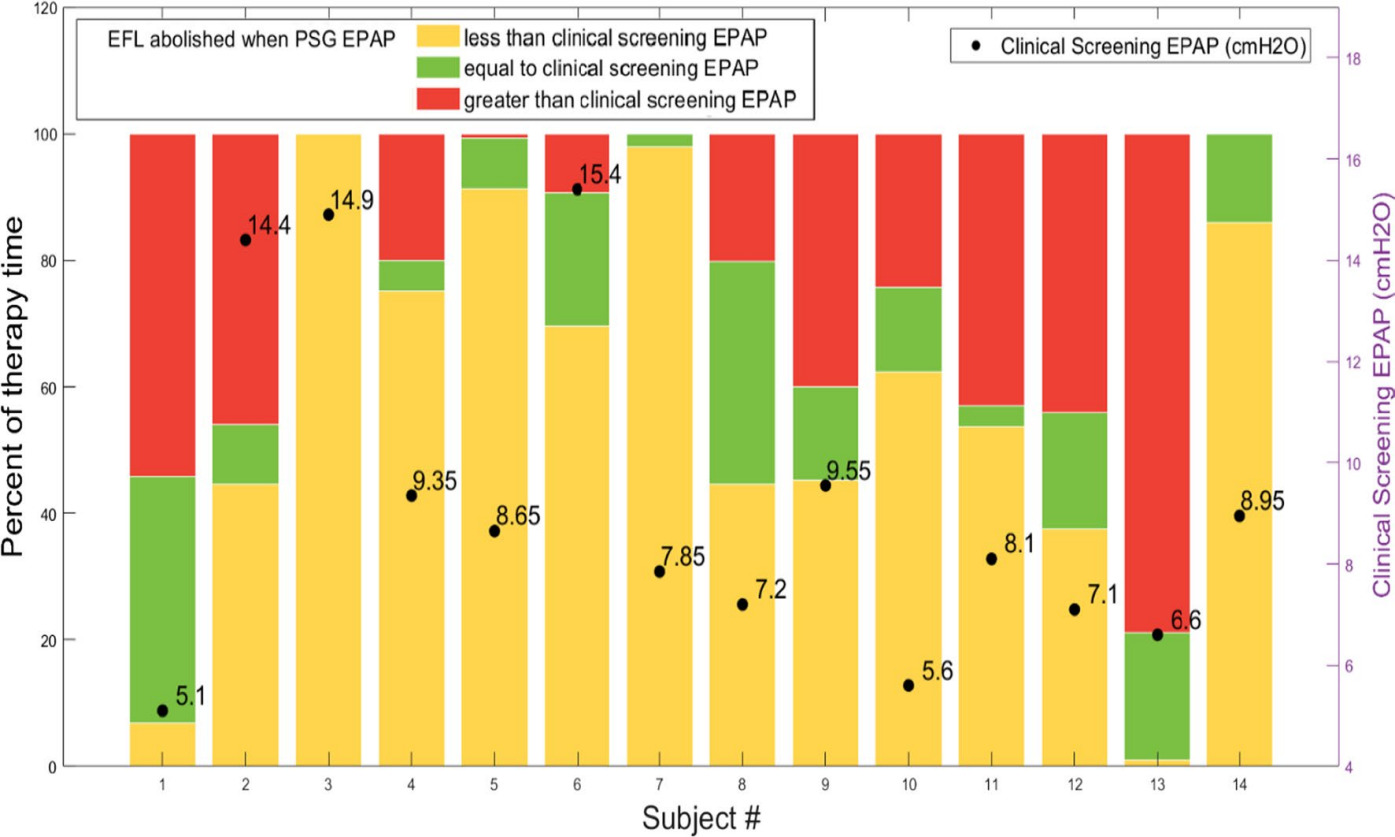
Percentage of time during PSG that the screening EPAP was equivalent to (green), greater than (yellow) or less than (red) that needed to eliminate EFL_T_ determined when supine awake Black circles and numbers represent the clinical EPAP screening value needed to abolish EFL_T_ (represented on the right-hand Y-axis). Overnight EPAP data were not available for participant 15 due to a data acquisition failure

Data for the whole group are shown in Fig. 3 which reflects the EPAP pressure determined during EFL_T_ screening (awake and supine) compared to the EPAP pressure determined by the ExpiraFlow technology during the single night overnight PSG. In these participants, on average, only 14.6% of sleep time was within ± 1 cmH_2_O of the screening pressure. Fifty-seven percent (8/14) of participants spent more than 50% of their sleep time below their screening EPAP pressure. Approximately 43% (6/14) of participants spent more than 40% of their sleep time above the screening pressure.

Table 2 represents total sleep time (TST) and sleep efficiency for overnight sleep study participants. Total sleep time averaged 220 min during the overnight trial with11 sleeping over 180 min. Due to technical issues, PSG data for participant 4 were not available. Sleep efficiency ranged between 2 and 91%.

The relationship between overnight EPAP and sleep stage was analyzed in 14 of the 15 participants who completed the overnight PSG. From the linear regression trending, four participants experienced an increased median EPAP, and seven experienced a decrease of median EPAP as sleep stage intensity progressed from N1 to REM; three did not show enough EPAP variability to identify trends related to sleep stage.

The relationship between overnight body position and EPAP were analyzed in 9 of the 15 participants who had a body position sensor attached during the PSG. On average, these participants spent 54% of the night in the supine position, 22% of the night on their right side, and 21% on their left side. Although EFL_T_ varied as body position changed, the results were not uniform for all participants. Additionally, no relationship was seen between disease severity, whether expressed as FEV_1_ or FEV_1_/FVC ratio and EPAP variability in the single-night study.

### 2-Week in-home study

Ten participants, 4 of whom were ventilator naïve, completed the two week in-home study. Data in Table 3 show the average maximum EPAP value and the average EPAP used for 90% of the study nights for each participant over the 2 weeks of home use. The variability in EPAP pressure in response to variable EFL_T_ is seen by the differences between these average maximum EPAP values for each participant, as well the aggregates for all participants—mean (SD) EPAP (6.4 (1.3) mH_2_O), the 90% EPAP (9.3 (3.2) mH_2_O), and the maximum EPAP (11.7 (3.4) cmH_2_O). A detailed example of this variability is provided in the data supplement (Additional file 1: Table S1, Fig. S1). There was an inverse relationship between the average EPAP and the FEV_1_/FVC (r = − 0.68, p = 0.031) and to a lesser degree an inverse relationship between average maximum EPAP and FEV1 (r = − 0.55, p = 0.103) of the participants. A moderate relationship was seen between BMI and mean EPAP (r = 0.59, p = 0.072).

**Table 3:** Average EPAP, average max EPAP and average 90% EPAP for all 2-week in-home study participants—see text for definition of terms

Patient ID	Starting EPAP	Average Max EPAP	Average EPAP	Average 90% EPAP
1	4.0	12.8	5.2	12.4
2	4.0	10.5	5	10.3
8	4.0	12.1	6.8	9.7
9	4.0	12.2	7.5	10.6
10	4.0	12.5	6.6	7.2
11	4.0	14.7	5.6	8.6
12	4.0	6.7	5.3	5.9
13	4.0	17.1	7.5	16.4
14	4.0	8.1	7.9	7.9
16	4.0	10.4	4.3	7.9
Avg	4.0	11.7	6.4	9.3
Std Dev	0.0	3.4	1.3	3.2

Expiratory positive airway pressure is reported in cmH_2_O

**Fig. 4:**
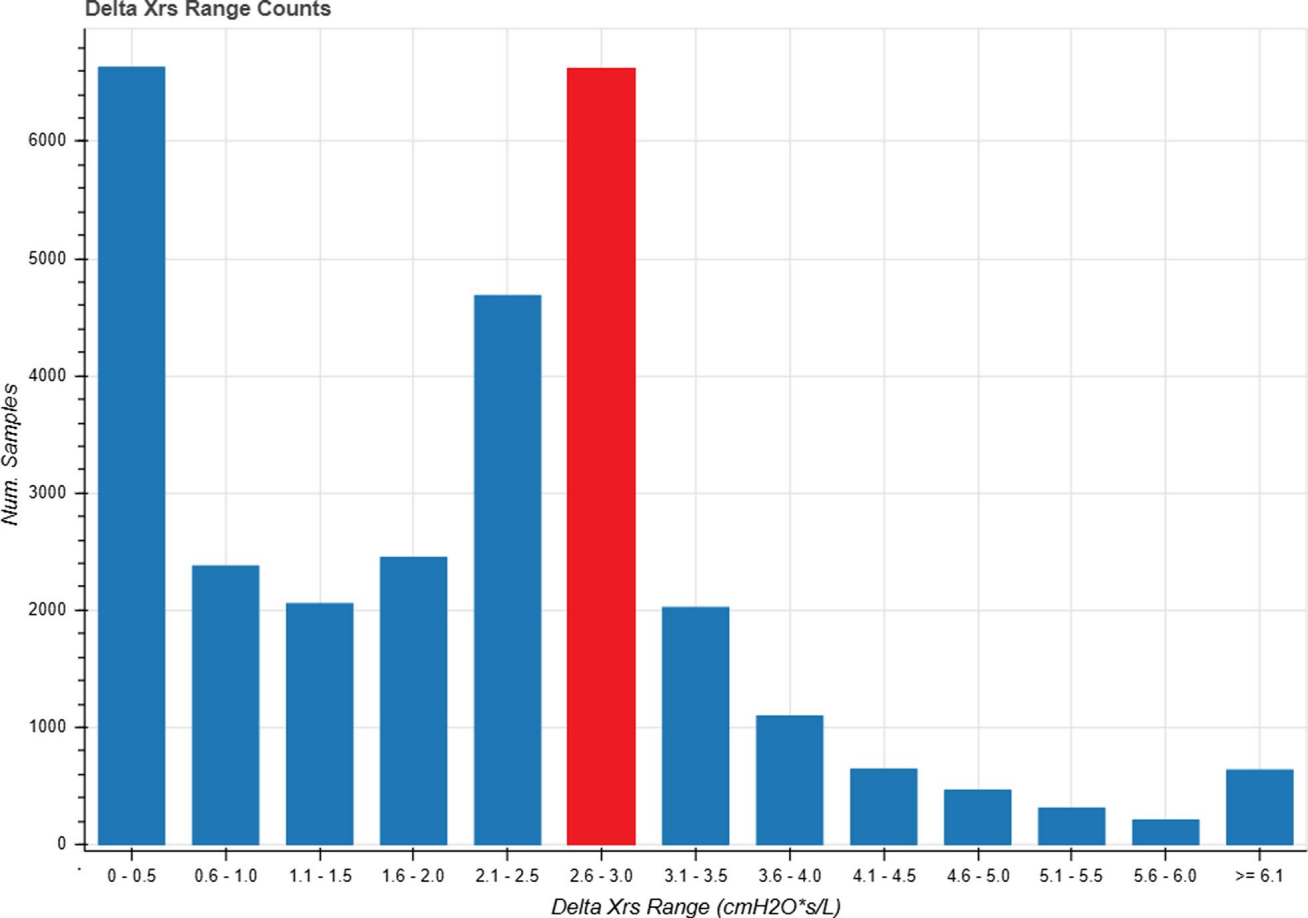
Population histogram showing the number of samples across all participants (n = 10) that fall in each range, relative to the threshold of expiratory flow limitation (red) Samples represent the mean ΔXrs in a 2 min period during ventilator use

Figure 4 shows the distribution of ∆Xrs for all participants (77.3% ≤ 2.8 cmH_2_O/L/s) during their 2-week in-home use. The auto-titration algorithm's effectiveness is shown in Table 4 where the measured minimum, maximum and meanattained ΔXrs values for all participants who completed the 2-week in-home study are presented. The percentage of all values at or below the threshold over the 2-week study period is shown in this table. The percentage of all ∆Xrs ≤ 2.8 cmH_2_O/L/s for all 2-week in-home use participants was 77.3%. In 7 of the 10 patients flow limitation was abolished for 80–100% of the night but this was not true for 2 subjects with poor ventilator adherence who contributed most of the flow limited data in Fig. 4. ∆Xrs values > 2.8 were quickly remedied with a mean time to return ∆Xrs ≤ 2.8 cmH_2_O/L/s of 5.91 min.

**Table 4:** Minimum, maximum and average attained ΔXrs value for all participants from the 2-week in-home ventilator use

Participant #	Min ΔXrs	Max ΔXrs	Avg ΔXrs	Percent ΔXrs < = 2.8
1	0.0	16.0	2.8	62.8%
2	0.0	11.6	2.1	77.1%
8	0.5	7.3	2.7	72.8%
9	0.0	20.7	3.8	49.2%
10	0.0	6.8	1.3	88.4%
11	0.0	8.7	2.2	87.0%
12	0.0	7.0	1.9	73.1%
13	0.0	19.8	3.2	47.9%
14	0.0	7.5	0.6	99.7%
16	0.0	13.9	1.97	74.7%

The percent ΔXrs <  = 2.8 column shows the percentage of all values at or below the threshold over the 2-week study period

Table 5 shows therapy compliance and comfort assessment of each participant in the in-home trial. Naïve participants averaged 3.2 (minimum 1.6, maximum 5.4) hours of daily use with 14-day compliance rate of 57%. NPPV users averaged 8.4 h (min 3.03, max 16.3) of daily use with an average compliance rate of 91.4%. Three out of 4 NPPV users rated FOT therapy very comfortable over their prior NPPV. Survey data were missing for 2 participants. Table 6 breaks down aggregate data for naïve and prior NPPV user groups. NPPV user group showed 8.5 h, while naïve group showed 2.5 h. of average nightly therapy use of the device. Altogether with 10 participants, 100 days with 692.4 h. of therapy usage was documented over the two weeks of in-home use.

**Table 5:** Therapy compliance and comfort assessment

Participant ID	Existing NPPV	Compliance %	Average night use (h)	NPPV Vs. FOT therapy rating
1	No	100	2.8	N/A–Naïve
2	No	57	1.6	N/A–Naïve
8	No	36	5.4	N/A–Naïve
9	Yes	86	4.3	Very comfortable
10	Yes	100	7.9	Very comfortable
11	Yes	100	10.2	Very comfortable
12	No	36	2.9	N/A–Naïve
13	Yes	100	16.3	Not reported
14	Yes	71	3.03	Uncomfortable
16	Yes	0	0	Value missing

**Table 6:** Aggregate therapy compliance and comfort assessment

Aggregate	Naïve group	NPPV group	Combined
Combined days of use	41	69	110
Total hours of therapy use	103.41	589.00	692.41
Average nightly hours of therapy use	2.52	8.54	6.29

## Discussion

In this study, we have used a new NIV therapy mode that screens and automatically detects EFL_T_ breath by breath using FOT, and dynamically adjusts EPAP to abolish EFL_T_ in a population of mild to severe COPD participants. Our principle aim was to investigate whether EFL_T_ in patients with COPD considered for maintenance ventilation varied during sleep and to assess the acceptability of abolishing EFL_T_ over a 2 week period. Our main findings were that: (1) EFL_T_ was highly variable within and between participants, as seen in overnight studies and over 2-week in-home device use, (2) it was possible to abolish EFL_T_ over multiple nights by automatic EPAP adjustment in real time, (3) in overnight PSG studies, on average less than 15% of sleep time was within ± 1 cmH_2_O of the screening pressure, (4) sleep quality with the ventilator system was acceptable with an average total sleep time of 220 min, and 11 out of 14 participants sleeping more than 180 min, (5) no direct relationships could be discerned between overnight EPAP and sleep staging or body positioning, and (6) average EPAP for 2-week in-home use participants differed from average 90% EPAP as well as average max EPAP. These data have implications for the way in which we apply non-invasive ventilation to clinically stable COPD patients.

Until recently, it was difficult to determine whether an individual breath was flow limited, let alone multiple breaths. The development of standardized methodologies using the forced oscillation technique [22] and the recognition that the within-breath change in low-frequency respiratory system reactance was highly correlated with more invasive methods of determining the presence of EFL_T_ during noninvasive ventilation [17] has transformed this field. We used the novel ExpiraFlow technology to determine dynamically the presence of EFL_T_ and the amount of EPAP required to overcome it. Like others [5, 6, 23] we found that substantially more of our COPD patients developed EFL_T_ when supine than erect. Our estimate of the presence of EFL_T_ is more conservative than other systems as there was 3 cmH_2_O of EPAP present in our ventilator circuit to ensure its effective operation. Moreover, we selected patients who required a significant amount of EPAP to abolish EFL_T_ during supine wakefulness. Nonetheless, we saw substantial within and between patient variation in EFL_T_, a finding that emphasizes that tidal flow limitation in COPD is not a fixed state but one that changes dynamically with body position and during sleep.

The main effect of postural change on EFL_T_ is mediated by a fall in end-expiratory lung volume [6, 23] something that also occurs in a state-dependent way during sleep [24]. Our data showed marked within night variation in the need for additional EPAP to abolish EFL_T_, although the reasons for this are likely to be multifactorial. While the degree of resting airflow obstruction and the body mass index of the patient are potentially important predictors, our single night study group was too small and heterogeneous to confirm this suspicion. A clearer relationship between lung function and the average EPAP needed to abolish EFL_T_ was seen in the 2-week data, although the degree to which this varied through the night was not related to waking lung function. State-dependent changes in minute ventilation and breathing pattern are also relevant and may explain why some subjects no longer exhibited EFL_T_ when asleep even though it was present during wakefulness. Finally, most of our patients were identified because they developed EFLT when supine. This selection criteria may explain why there was no clear association between posture and EFLT during sleep as the pressure required to overcome flow-limitation related to posture change had already been established. More detailed investigation of these issues across a wider range of COPD patients is needed.

Expiratory flow limitation is an important factor influencing the success of invasive ventilation [12]. Although adjustments in respiratory timing can abolish this when breathing frequency is controlled, only an increase in EPAP can do this during noninvasive ventilation and this must be done without the risk of a consequential increase in end-expiratory lung volume. This is possible using ExpiraFlow technology and is associated with a decrease in respiratory drive measured using the parasternal EMG [14]. Our data extend these observations to patients using this equipment overnight. Sleep quality in COPD patients has been known to be poor for many years [25] and the PSG findings in our single night studies were similar to those in less instrumented patients [26].This was true even though the amount of EPAP applied to abolish EFL_T_ increased to 19 cmH2 in some patients. Although the mean EPAP in both the single night and 2-week studies was similar to that anticipated from the awake EPAP setting, this value did not predict the amount of EPAP needed to abolish EFL_T_ consistently overnight.

Data from the period of extended use at home showed that EFL_T_ was successfully abolished in most patients. Overall treatment adherence was good, with over 6 h of overnight ventilator use in the 2 weeks of study. However adherence was better in patients who had experience of domiciliary NIV, some of whom used the equipment during waking hours as well. A single night cross over study has suggested that abolishing EFL_T_ reduces the number of prematurely initiated breaths during NIV [18]. Other data have shown that neck muscle inspiratory activation during sleep is a response to nocturnal hyperinflation and impairs sleep quality [27]. Our data are compatible with a beneficial effect on sleep and treatment adherence by limiting the degree of hyperinflation and sleep disturbance which may underpin the acceptability of treatment. Future studies should explore this possibility.

This observational study has strengths and weaknesses. We identified a subgroup of participants in whom EFL_T_ was present when supine and awake. We considered patients attending for initiation or monitoring of treatment, thereby increasing the generalizability of our findings in daily practice. As expected, our volunteers were a mixture of pure COPD and COPD-OSA overlap patients with 2 patients no longer meeting the COPD spirometry criteria when tested before our study. We included their data on an intention to treat basis. We did not see significant differences in the EPAP requirements or the acceptability of ventilatory support between any of these subgroups. However, our data are still best seen as hypothesis-generating. The participants recruited were not severely hypoxemic, and we did not monitor transcutaneous CO_2_ during sleep or morning arterial blood gas tensions which may have improved with more effective ventilation-perfusion matching across a less flow-limited lung. We did not anticipate the degree to which EFL_T_ would change overnight and so more detailed analysis of the degree of the physiological determinants of this process were not incorporated in the study. However, we have identified several areas where further mechanistic investigations are required. We successfully abolished EFL_T_ on multiple nights in a 2-week period but not for 100% of the night. In part this reflects the algorithm we used which adjusts EPAP pressure on a breath-by-breath basis until flow limitation is abolished and hence it takes some time after a change for unrestricted airflow to be restored. However, this was done within 3–6 min on average without changing the participant’s perceived sleep quality.

## Conclusions

Our study has highlighted the spontaneous fluctuation of tidal expiratory flow limitation during sleep, which could contribute to sleep disruption in COPD patients. This can be abolished by the automated application of carefully tailored amounts of EPAP. We have shown that this is both technically possible and clinically acceptable offering the prospect of more physiologically specific forms of noninvasive ventilatory support in spontaneously breathing COPD patients. These proved to be effective and relatively well tolerated with regular use over an extended period of time.

## Supplementary Information

**Additional file 1: Table S1.** Representative case of one participant (participant 11) daily EPAP parameters over 2-weeks. **Fig S1.** Subject 11—DeltaXrs samples for every session (n = 14) for a typical participant (subject 11), where the start of each session treated as time = 0. Each point represents a 2 min average of the ∆Xrs values occurring during that time.

## Data Availability

Data available on application to the authors.

## Abbreviations

BMI: Body mass index
COPD: Chronic obstructive pulmonary disease
CPAP: Continuous positive airway pressure
DH: Dynamic hyperinflation
EFL_T_: Tidal expiratory flow limitation
FEV1: Forced expiratory volume in the 1st second
FVC: Forced vital capacity
EPAP: Expiratory positive airway pressure
PEEP: Positive end expiratory pressure
PEEPi: Intrinsic positive end expiratory pressure
PEEPopt: Auto-titrated optimal positive end expiratory pressure
FOT: Forced oscillation technique
NIV: Noninvasive ventilation
NPPV: Noninvasive positive pressure ventilation
WOB: Work of breathing
ΔXrs: Difference between the average Xrs measured during inspiration and the one measured during expiration
PSG: Polysomnography
S/T: Spontaneous/timed NIV mode
O/N: Overnight
