# Supplementary material for: Overnight variation in tidal expiratory flow limitation in COPD patients and its correction: an observational study
Source: Respir Res. 2021 Dec 23;22:319. doi: 10.1186/s12931-021-01913-7 (PMC8697433; doi:10.1186/s12931-021-01913-7)
Supplement: Supplementary file 1 — Additional file 1: Table S1. Representative case of one participant (participant 11) daily EPAP parameters over 2-weeks. Fig S1. Subject 11—DeltaXrs samples for every session (n = 14) for a typical participant (subject 11), where the start of each session treated as time = 0. Each point represents a 2 min average of the ∆Xrs values occurring during that time. [file 12931_2021_1913_MOESM1_ESM.docx]

## Supplement to A40 paper

**A Entry criteria**

## Inclusion Criteria

1. Age > 40 years of age; < 80 years of age
2. Ability to provide informed consent
3. Diagnosis of COPD and either using or being considered for non-invasive ventilatory support
4. Must be able to maintain SpO2 greater than 88% at rest and during EPAP titration

## Exclusion Criteria:

1. Any major non COPD uncontrolled disease or condition, such as congestive

heart failure, malignancy, liver or renal insufficiency (that requires current

evaluation for liver or renal transplantation or dialysis), amyotrophic lateral

sclerosis, or severe stroke, or other condition as deemed appropriate by

investigator as determined by review of medical history and / or participant

reported medical history

1. Suffering from a COPD exacerbation at the time of data collection or in the 30

days prior to data collection

1. Self-reported Pregnancy
2. Employee or family member that is affiliated with Philips Respironics
3. Currently employed by a manufacturer of respiratory products or family member employed by a manufacturer of respiratory products
4. History of bullous emphysema
5. History of pneumothorax
6. Evidence of acute sinusitis or otitis media
7. Hypotension
8. Participants at risk for aspiration of gastric contents
9. Epistaxis
10. Participants in respiratory failure
11. Inability to maintain a patent airway or adequately clear secretions

Additional Inclusion/Exclusion Criteria for the 2 week treatment at home phase:

Inclusion Criteria:

1. Participants who completed the initial study and who would be willing to use the Vector NIV device at home during the night for a 2 week period ***OR***
2. Participants prescribed and currently using a PAP or NIV device at home who meet study inclusion/exclusion criteria of primary protocol

Exclusion Criteria

1. Participants currently using a PAP or NIV device at home with a documented EPAP setting on their current device that is greater than then the mean or final EPAP determined during the therapy session of the screening visit.

**Additional information about the ventilator system**

The algorithm used to identify EFLt was validated using a bench test setup consisting of different combinations of calibrated linear resistors and various fixed volume containers to simulate respiratory system resistance and compliance.  The load impedances were measured with another device designed to measure ΔXrs (Resmon ProTM, ResTech, USA) in order to provide the target ΔXrs values to which the ExpiraFlow algorithm was benchmarked. The Resmon Pro  uses FOT to measure lung function during tidal breathing by analyzing the respiratory system’s response to pressure pulsations to detect expiratory flow limitation (EFLt) using the same patented method described by Dellaca.

In the screening mode, up to 5 minutes of data were collected from the patient while wearing an oro-nasal mask to ensure that between 5 and 20 technically satisfactory breaths were obtained against a fixed background EPAP of 3.0 cmH_2_O. Breaths were automatically excluded if either the calculated inspiratory or expiratory duration was 0.5 sec or less or the calculated system resistance exceeded 40 cmH_2_O/L/sec. Additionally breaths are not analysed if there is evidence of excessive leak when they occur. As leak changes as pressure increases, the device uses a pressure/flow curve to determine excessive flow for a given pressure.  If the flow is above that value for a given pressure, the leak is considered to be excessive and the breath is not used in the calculation.

In the therapy mode, flow-limitation is assessed continuously on a breath by breath basis with a starting EPAP value of 4 cmH_2_O. If the value of deltaXrs exceeds 2.8 cmH_2_O /L/Sec then the EPAP is increased by 0.1 cmH_2_0 until flow limitation is abolished. At this stage EPAP is decreased by a similar amount until flow limitation returns or the value of 4cmH_2_O of EPAP is achieved.

Table S1 represents the variability of average, average max and average 90% EPAP seen in an individual participant each day of the 2-week in-home use period. The average EPAP (5.6) is 65% of the 90% EPAP (8.6), implying EPAP variability in response to the variable flow limitation. Figure 4a presents the corresponding ∆Xrs values for the same participant over the 2-week in home device use.

| Day | **1** | **2** | **3** | **4** | **5** | **6** | **7** | **8** | **9** | **10** | **11** | **12** | **13** | **14** | **MEAN** | **Std Dev** |
| --- | --- | --- | --- | --- | --- | --- | --- | --- | --- | --- | --- | --- | --- | --- | --- | --- |
| Starting EPAP | 4 | 4 | 4 | 4 | 4 | 4 | 4 | 4 | 4 | 4 | 4 | 4 | 4 | 4 | 4 | 0.0 |
| Max EPAP | 15.4 | 14.1 | 19.7 | 12.8 | 14.5 | 10.9 | 12.3 | 12.3 | 13.3 | 15.6 | 15.1 | 16.2 | 19.2 | 14.3 | 14.7 | 2.5 |
| Avg EPAP | 5.7 | 5.3 | 5.1 | 6.1 | 6 | 4.5 | 6.1 | 5.7 | 5.4 | 5.3 | 5.2 | 6.2 | 5.5 | 6.4 | 5.6 | 0.5 |
| 90% EPAP | 8.82 | 7.6 | 7.6 | 9.6 | 9.2 | 5.5 | 9.6 | 8.5 | 9.6 | 7.2 | 7.6 | 9.8 | 8.8 | 11.3 | 8.6 | 1.4 |

Table S1 : Representative case of one participant (participant 11) daily EPAP parameters over 2-weeks

Fig. S1 shows the frequency of samples with EFL_T_ (above dotted line) and without (below dotted line), for a typical participant over their 2-week in-home ventilator use. The dotted black line represents ∆Xrs = 2.8. As can be seen, ∆Xrs tends to cluster around 2.8 threshold, as would be expected when EFL_T_ is abolished.

**
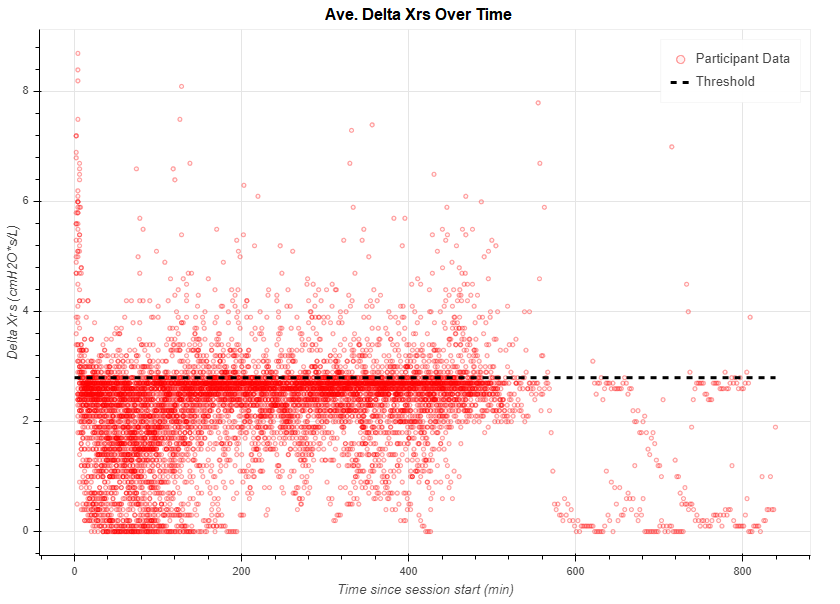
**

Fig S1: Subject 11 - DeltaXrs samples for every session (n=14) for a typical participant (subject 11), where the start of each session treated as time = 0. Each point represents a 2 minute average of the ∆Xrs values occurring during that time.
